# Airborne Transmission of Highly Pathogenic Influenza Virus during Processing of Infected Poultry

**DOI:** 10.3201/eid2311.170672

**Published:** 2017-11

**Authors:** Kateri Bertran, Charles Balzli, Yong-Kuk Kwon, Terrence M. Tumpey, Andrew Clark, David E. Swayne

**Affiliations:** US Department of Agriculture, Athens, Georgia, USA (K. Bertran, C. Balzli, Y.-K. Kwon, D.E. Swayne);; Centers for Disease Control and Prevention, Atlanta, Georgia, USA (T.M. Tumpey);; International Veterinary Consultant, Pendleton, Oregon, USA (A. Clark)

**Keywords:** H5N1 highly pathogenic avian influenza, poultry, airborne transmission, aerosol, droplet, viruses

## Abstract

Exposure to infected poultry is a suspected cause of avian influenza (H5N1) virus infections in humans. We detected infectious droplets and aerosols during laboratory-simulated processing of asymptomatic chickens infected with human- (clades 1 and 2.2.1) and avian- (clades 1.1, 2.2, and 2.1) origin H5N1 viruses. We detected fewer airborne infectious particles in simulated processing of infected ducks. Influenza virus–naive chickens and ferrets exposed to the air space in which virus-infected chickens were processed became infected and died, suggesting that the slaughter of infected chickens is an efficient source of airborne virus that can infect birds and mammals. We did not detect consistent infections in ducks and ferrets exposed to the air space in which virus-infected ducks were processed. Our results support the hypothesis that airborne transmission of HPAI viruses can occur among poultry and from poultry to humans during home or live-poultry market slaughter of infected poultry.

Since 2003, approximately 850 human cases of Eurasian A/goose/Guangdong/1/1996 (Gs/GD) lineage H5N1 virus infection have been reported; case-fatality rate is 53% (*1**–**3*). Most human infections with highly pathogenic avian influenza (HPAI) subtype H5N1 virus have occurred following direct or indirect exposure to infected poultry in live-poultry markets (LPM) in developing countries (*1**–**3*). The main risk factors associated with human infections include visiting an LPM or performing activities with intensive contact with infected poultry, like slaughtering, defeathering, or preparing poultry for cooking (*3**,**4*).

Poultry-to-human avian influenza (AI) virus transmission can occur from 3 types of exposure: fomite-contact transmission, including contact with contaminated surfaces; droplet transmission, in which large (>5 μm) particles contact a person’s conjunctiva or respiratory mucosa; and droplet nuclei transmission (or aerosol transmission), in which a person inhales small (<5 μm) particles suspended in the air (*5**–**8*). The LPM setting plays a critical role in maintaining, amplifying, and disseminating AI viruses among poultry and from poultry to humans (*1**,**2**,**9*), with indirect evidence of potential transmission via fomites, as supported by the detection of AI viruses in the environment (*10**–**12*), and airborne exposure, supported by the recent isolation of influenza A viruses from air sampled at LPMs in China (*12*). Furthermore, viable AI viruses can be detected in the air where live poultry are kept and processing activities, such as slaughtering and defeathering, are performed (*12*).

Collective epidemiologic and surveillance data suggest that the slaughter of infected poultry is a major public health concern. In our study, we determined that viable airborne HPAI virus particles were generated during simulated processing of HPAI virus–infected poultry and that the airborne virus was transmitted to virus-naive poultry and mammals.

## Materials and Methods

### Viruses

Eurasian goose/Guangdong lineage H5N1 viruses were selected from human cases of influenza A(H5N1) virus, representing various years, hosts, countries, and clades (*1**,**3*) (Table 1). For experiment 1, we used 7 viruses (Table 1, all but Mong/05) for challenge in chickens, of which 4 that generated airborne virus particles were used in ducks. For experiment 2, we used Mong/05 and VN/04 viruses as challenge viruses. We propagated and titrated the viruses in embryonating chicken eggs (ECE) by standard methods (*13*).

**Table 1:** Information on Eurasian A/goose/Guangdong/1/1996 lineage (H5N1) virus isolates used in this study

Isolate	Abbreviation	Country	Host/source	Genetic clade	Accession nos.*
A/Vietnam/1203/2004	VN/04	Vietnam	Human	1	HM006756–63
A/chicken/Vietnam/NCVD-878/2011	VN/878/11	Vietnam	Poultry	1.1	Not available
A/chicken/West Java-Subang/29/2007	WJ/07	West Java	Poultry	2.1.3	EPI533441†
A/whooper swan/Mongolia/244/2005	Mong/05	Mongolia	Water fowl	2.2	GU186700–07
A/chicken/Egypt/102d/2010	Eg/10	Egypt	Poultry	2.2.1	HQ198270.1
					HQ908480.1
					KR732432.1
					KR732440.1
					KR732445.1
					KR732492.1
					KR732530.1
A/Egypt/N6658/2011	Eg/11	Egypt	Human	2.2.1	EPI372860–67†
A/chicken/Vietnam/NCVD-675/2011	VN/675/11	Vietnam	Poultry	2.3.2.1	KR732403
					KR732406
					KR732415
					KR732468
					KR732481
					KR732506
					KR732521
					KR732536
A/chicken/Vietnam/093/2008	VN/08	Vietnam	Poultry	7.2	FJ538949.1
					FJ538950.1
					FJ842480.1

*Accession numbers from GenBank except as indicated. Accession numbers represent sequences from all available segments of influenza A virus.  †Accession number from GISAID (http://platform.gisaid.org).

### Animals

For experiment 1, we obtained 9-week-old specific pathogen free (SPF) white Leghorn chickens (*Gallus domesticus* from the US Department of Agriculture Southeast Poultry Research Laboratory, Athens, GA, USA) and 8-week-old domestic Pekin ducks (*Anas platyrhynchos domestica*, from McMurray Hatchery, Webster City, IA, USA). All birds were serologically negative for influenza A virus infection by hemagglutinin inhibition (HI) test (*13*) before inoculation. For experiment 2, chickens and ducks were used as either infected or virus-naive exposed birds. Intravenous injection of sodium pentobarbital (100 mg/kg) was used to euthanize naive exposed survivors. Naive 3- to 5-month-old female domestic ferrets (*Mustela putorius furo*; Marshall BioResources, North Rose, NY, USA, and Triple F Farms Inc., Sayre, PA, USA) were used as the mammalian model for HPAI virus transmission to humans (*4*). Ferrets were anesthetized with an intramuscular injection of a mixture of ketamine (25 mg/kg), xylazine (2 mg/kg), and atropine (0.05 mg/kg) before nasal sample collection or euthanasia by intracardiac injection of sodium pentobarbital. Ferrets were H5-seronegative by HI test and virus neutralization test, and nasal wash samples were negative for virus isolation in ECE before exposure. All procedures were performed in accordance with protocols approved by the Institutional Animal Care and Use Committee and the Institutional Biosecurity Committee.

### Environmental Conditions in the Processing Enclosure

**Figure 1:**
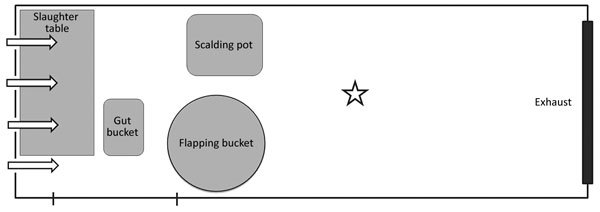
Processing area for study of airborne transmission of highly pathogenic influenza virus during processing of infected poultry. The star represents the location of the air sampler (experiment 1) or the naive hosts (experiment 2). The arrows indicate the airflow within the HEPA enclosure. The enclosure was 1.5 m wide × 6.7 m long × 2.1 m high, with 8.3 air changes/h (340 m^3^/h) and a velocity of 0.046 m/s.

All experiments were conducted in Biosafety Level 3 animal facilities enhanced with additional biosafety features. The processing area was a high-efficiency particulate air (HEPA) enclosure (Class Biologically Clean Ltd., Madison, WI, USA) 1.5 m wide × 6.7 m long × 2.1 m high with unidirectional and single-pass airflow of 8.3 air changes/h (340 m^3^/h) at 0.046 m/s from the processing area toward the air samplers or the naive animals (Figure 1). The mean temperature in the enclosure during the slaughter runs was 24.2°C ± 0.4°C; mean relative humidity was 81.0% ± 1.7%. We performed all procedures using adequate personal protective equipment: respiratory protection (HEPA-filtered powered air purifying respirators with full-shroud shield), closed-front gown, double gloves, and rubber boots.

### Air Sampling

The National Institute for Occupational Safety and Health (NIOSH) cyclone air sampler (model BC 251; NIOSH, Morgantown, WV, USA) collected particles and sorted them by their aerodynamic diameters into >4 µm, 1–4 µm, and <1 µm fractions at a flow rate of 0.0035 m^3^/min (*14*). We mounted 2 stationary samplers 1.2 m above ground, 1 within the enclosure 80 cm downwind from the processing area (center to center) and the other outside the enclosure as negative control. Samplers were operated for the duration of each slaughter run plus 10 minutes; we sampled a total of 0.158–0.280 m^3^ air per study trial, depending on the number of birds processed per trial.

### Experimental Design

#### Experiment 1: Generation of Airborne HPAI Virus Particles during Simulated Processing of Infected Poultry

Each run (i.e., tested virus per bird species) was repeated at least twice for reproducibility. Chickens (10 for VN/04 and 5 for all other viruses) and ducks (5 per virus) were inoculated intranasally with 10^5.3^–10^6.5^ mean egg infectious dose (EID_50_)0.1 mL per virus and housed in negative-pressure isolators with HEPA-filtered ventilation. We moved chickens at 24 h after inoculation and ducks at 2.5 days after inoculation, which corresponded to times of peak shedding titers, to the processing enclosure while they were still asymptomatic. We anesthetized them by intramuscular injection of ketamine (10 g/kg) and xylazine (1 g/kg) and collected oral swab samples to confirm infection. The anesthetized birds were processed following 5 steps (total duration 6–7 min/bird) (*15*): 1) manual killing by severing the right jugular vein with a scalpel blade, causing bleeding and agonal involuntary muscle contractions (1 min); 2) scalding in a covered pot (52–53°C/2 min); 3) manual defeathering (2 min); 4) evisceration and removal of head, feet, and internal organs (1.5 min); and 5) cleanup of processing area with water (0.5 min). We rubbed the ducks with detergent before the scalding step to remove preening oils and facilitate defeathering. During the processing, air samplers were used as aforementioned. After each run, we disinfected all materials and surfaces within the enclosure, as well as the units holding the infected birds, with Virkon S 2% (DuPont, Wilmington, DE, USA). We tested swab samples for viable virus in ECE and titrated aerosol samples in ECE (*16*). The minimum detectable titer in ECE was 0.9 log_10_ EID_50_/mL.

#### Experiment 2: Transmission of HPAI Viruses to Poultry and Ferrets during Simulated Processing of Infected Poultry

We performed 5 runs (Table 2). We inoculated chickens and ducks intranasally with 10^5.9^–10^6.1^ EID_50_/0.1 mL per virus (Table 2) and housed them in negative-pressure isolators. As in experiment 1, we anesthetized asymptomatic chickens and ducks, took oral and cloacal swab samples, and processed the birds using the 5-step method. During the processing, naive chickens, ducks, or ferrets (Table 2) were placed in cages at the same location and height as the air samplers in experiment 1 (with variations in experiment 2.1). After completion of each run, we placed the exposed animals in negative-pressure isolators and monitored them for clinical signs for 2 weeks. We collected oral and cloacal swab samples from exposed chickens at time of death and from exposed ducks at 3, 7, 10, and 14 days postexposure (dpe). We collected nasal wash samples and bodyweight measures from exposed ferrets at 3 and 7 dpe. We euthanized ferrets that had lost > 25% bodyweight or exhibited neurologic dysfunction. We performed necropsies on dead or euthanized exposed animals and collected tissues in 10% buffered formalin for hematoxylin/eosin and immunohistochemical staining (*17*). We titrated swab and nasal wash samples in ECE (*16*). At 14 dpe, we collected blood from the survivors for homologous HI and virus neutralization testing, then euthanized them.

**Table 2:** Experimental design and clinical outcome of animal hosts exposed to airborne H5N1 HPAI viruses through simulated live-poultry market slaughter*

Virus	Intranasally infected birds processed (no.)	Duration of slaughter process, min	Naive exposed hosts (no.)†	Deaths of exposed hosts (mean time of death)	Virus detection in exposed hosts‡	Seroconversion in surviving exposed hosts§
Mong/05	Chickens (10)	60	Chickens (5)	5/5 (4.4 dpe)	5/5 at time of death¶	NA
VN/04	Chickens (10)	60	Chickens (5)	5/5 (4.0 dpe)	5/5 at time of death¶	NA
VN/04	Chickens (10)	60	Ferrets (4)	3/4 (8.3 dpe)	1/4 on 3 dpe (3.0)¶	0/1
VN/04	Ducks (5)	30	Ducks (5)	0/5	5/5 (1.6)	1/5
VN/04	Ducks (5)	30	Ferrets (3)	0/3	0/3	0/3

*dpe, days postexposure; EID, mean egg infectious dose; Mong/05, A/whooper swan/Mongolia/244/2005; NA, not available; VN/04, A/Vietnam/1203/2004. †Exposed hosts placed 75–80 cm from the slaughter area. ‡No. positive/total no. Numbers in parentheses indicate mean virus titers (log_10_ EID_50_/mL) determined by virus isolation in embryonating chicken eggs from oral and cloacal swab samples of exposed poultry or by nasal wash samples of exposed ferrets. §Determined by hemagglutinin inhibition and virus neutralization tests when >12 dpe serum samples were available. ¶Virus antigen was detected by immunohistochemistry in tissues of 5/5 Mong/05-exposed chickens, 5/5 VN/04-exposed chickens, and 3/4 VN/04-exposed ferrets.

### Statistical Analysis

Using the D’Agostino-Pearson test, we determined that none of our parameters were normally distributed. We conducted 2-tailed Mann-Whitney test to determine significant difference in mean viral titers (p<0.05) using GraphPad Prism 6 (GraphPad Software, Inc., La Jolla, CA, USA). 

## Results

### Experiment 1

**Figure 2:**
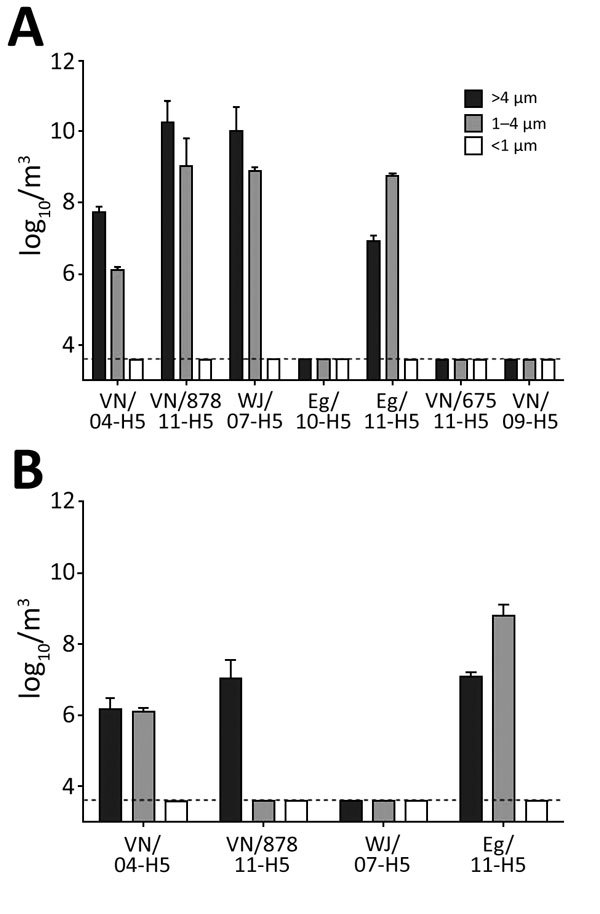
Highly pathogenic avian influenza virus isolation from air samples collected using cyclone air sampler during simulated slaughter of infected chickens (A) and ducks (B) in study of airborne transmission of highly pathogenic influenza virus during processing of infected poultry. Detection of virus was attempted in 3 different airborne particle sizes. Error bars indicate virus recovery from >2 repeats per run. Dashed lines indicate limit of detection by virus isolation of 3.6 log_10_ EID_50_/m^3^ air sampled. Isolate names are as given in Table 1.

Preslaughter swab samples were positive for virus in all asymptomatic birds with titers >1.5 log_10_ EID_50_/mL. We isolated VN/04, VN/878/11, WJ/07, and Eg/11 viruses from air samples collected when processing virus-infected chickens, with highest virus quantity in >4 µm particles, moderate quantities in 1–4 µm particles, and no virus in <1 µm particles. We did not detect Eg/10, VN/67511, or VN/09 viruses in air samples (Figure 2, panel A). We used these 4 airborne viruses recovered from the chicken study in the duck slaughter experiment; we detected VN/04 and Eg/11 viruses in both >4 µm and 1–4 μm particles, and VN/878/11 virus in >4 µm particles. We did not detect airborne virus from slaughter of WJ/07 virus–infected ducks (Figure 2, panel B). We detected no virus from aerosol samplers located outside the enclosure.

### Experiment 2

#### Experiment 2.1. Transmission of A/whooper swan/Mongolia/244/2005(H5N1) HPAI Virus to Naive Chickens Exposed during Simulated Processing of Infected Chickens

Swab samples were virus positive from asymptomatic Mong/05 virus–inoculated chickens. As a variation, for every 10 processed Mong/05 virus-infected chickens, we placed 5 exposed naive chickens 75 cm, 150 cm, or 300 cm from the slaughter area (all distances found in an LPM scenario) 1.2 m above ground, in a holding cage similar to those used in LPMs. Regardless of the distance from the processing area, all exposed chickens died between 3 and 6 dpe. All oral and cloacal swab samples collected at time of death were positive by virus isolation. We found lesions typical of those caused by HPAI and AI viral antigen in multiple internal organs of all exposed chickens, indicating infection after droplet/aerosol infection (Table 2).

#### Experiment 2.2. Transmission of A/Vietnam/1203/04(H5N1) HPAI Virus to Naive Chickens and Ferrets Exposed during Simulated Processing of Infected Chickens

**Figure 3:**
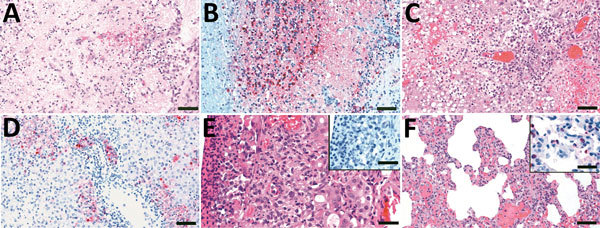
Histologic lesions and immunohistochemical detection of viral antigen in samples from ferrets exposed to live poultry market processing of highly pathogenic avian influenza A/Vietnam/1203/04 (H5N1) virus–infected chickens in study of airborne transmission of highly pathogenic influenza virus during processing of infected poultry. A) Olfactory bulb, 7 dpe, showing diffuse and severe neuropil malacia with mild cavitation and focal hemorrhages. Scale bar = 50 μm. B) Olfactory bulb, 7 dpe, showing viral antigen detected in neuropil, astrocytes, and neurons. Scale bar = 50 μm. C) Liver, 8 dpe, showing confluent coagulative necrosis of hepatocytes and bile duct necrosis with mononuclear cellular infiltrate in the portal triad. Scale bar = 50 μm. D) Liver, 8 dpe, showing viral antigen detected in hepatocytes, bile duct epithelia, and cellular debris. Scale bar = 50 μm. E) Nasal cavity, 7 dpe, showing moderate necrotic rhinitis with coagulative necrosis of mucous glandular epithelial cells; insert shows no viral antigen detected in mucosal membrane. Scale bars = 25 μm. F) Lung, 7 dpe, showing mild histiocytic interstitial pneumonia; insert shows viral antigen detected in type II pneumocytes. Scale bars = 25 μm. dpe, days postexposure.

Swab samples were virus positive from asymptomatic VN/04 virus–inoculated chickens. Following the processing of infected chickens, all 5 exposed naive chickens died between 3 and 5 dpe, and all oral and cloacal swab samples we collected at time of death were virus positive (Table 2). Out of 4 exposed ferrets, 2 died, 1 on 6 dpe and the other on 7 dpe; another ferret was euthanized on 12 dpe. Neurologic disease, with lesions typical of those caused by HPAI and AI virus in multiple internal organs including the brain, developed in these 3 ferrets (Figure 3). The ferret that died on 7 dpe had positive nasal wash samples collected at 3 dpe (3.0 log_10_ EID_50_/mL), and the ferret that was euthanized on 12 dpe seroconverted (Table 2). The survivor had no antibodies to AI or pathologic lesions and no virus in nasal wash samples, and it was the only ferret to gain weight; therefore, we considered it not infected. In summary, 3 of 4 naive ferrets became infected after droplet/aerosol exposure.

#### Experiment 2.3. Transmission of A/Vietnam/1203/04(H5N1) HPAI Virus to Naive Ducks and Ferrets Exposed during Simulated Processing of Infected Ducks

**Figure 4:**
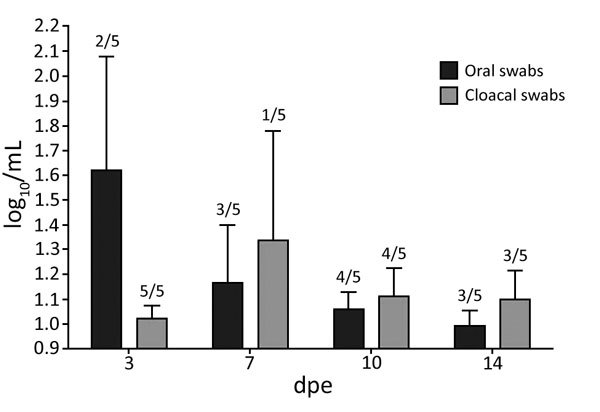
Virus titers in oral and cloacal samples of ducks exposed to simulated live poultry market slaughter of highly pathogenic avian influenza A/Vietnam/1203/04(H5N1) virus–infected ducks in study of airborne transmission of highly pathogenic influenza virus during processing of infected poultry. Shedding titers are expressed as log_10_ with error bars included. Numbers on top of the bars indicate the number of positive samples out of the 5 tested samples at each time point. The limit of detection was 0.9 log_10_ median egg infection dose/mL.

Swab samples were virus positive from asymptomatic VN/04 virus-inoculated ducks. Following the processing of infected ducks, exposed naive ducks and ferrets did not exhibit clinical signs nor did they die over the 2-week observation period (Table 2). We isolated virus from oral and cloacal samples of exposed ducks; peak individual titers were 3.1 log_10_ EID_50_/mL and mean titers were 1.6 log_10_ EID_50_/mL on 3 dpe (Figure 4). All exposed ferrets gained weight and had negative nasal wash samples and were considered to be uninfected (Table 2). All exposed ducks and ferrets were seronegative at termination with the exception of 1 duck (HI titer of 8) (Table 2). 

## Discussion

The epidemiology of human influenza A (H5N1) infections suggests that LPM slaughter processing of infected poultry could provide sufficient exposure to cause transmission to humans (*1**–**4*). Zhou et al. showed that viable H5, H7, and H9 AI viruses with human zoonotic potential are detectable in the air of LPMs in China (*12*). Here we demonstrated that the processing of asymptomatic HPAI virus–infected poultry in high biocontainment laboratory facilities produced airborne HPAI virus particles, which are airborne transmissible to naive poultry and mammals.

The simulated slaughter of infected poultry generated viable virus predominantly in droplets (>4 µm) and aerosols (1–4 µm) but none in particles (<1 µm). Our findings align with those of previous studies that used air samplers in LPMs (*12*) and swine barns (*18**,**19*), and farm-to-farm dissemination studies to demonstrate airborne virus (*20*). Determining the particle size distribution has key implications for the control of influenza in humans through droplet and aerosol transmission. Infectious particles with aerodynamic diameters <4 µm (i.e., aerosols) can more easily reach the lower respiratory tract of humans, where AI viruses with binding specificity for α-2,3 receptors primarily replicate, than larger particles can (*21*). The recovery efficiencies we obtained in this study (<10 log_10_ particles per m^3^ in >4 µm fraction) were higher than those from similar sampling methods in LPMs (*12*) possibly due to standardized high-dose challenge of all birds, optimized timing of slaughter, controlled environmental conditions, or other reasons. Human-origin viruses of clades 1 and 2.2.1 and avian-origin viruses of clades 1.1, 2.2, and 2.1.3 were detected in droplets and aerosols during the slaughter of infected chickens. However, other poultry-origin viruses (clades 2.2.1, 2.3.2.1, and 7.2) were not detectable (Figure 2). Three viruses (VN/04, VN/878/11, and Eg/11) generated consistent infectious droplets, aerosols, or both during the slaughter of infected ducks (Figure 2). Our results suggest that differences in the potential for incorporation of infectious HPAI viruses in airborne particles generated while processing infected poultry vary with the infected poultry species and specific HPAI virus. This study aimed to detect infectious virus; whether the viruses that were not detected or transmitted were not aerosolized, or whether they were present in airborne particles but were not infectious, warrants further study.

The processing of HPAI virus–infected chickens seems to be more effective at generating infectious droplets and aerosols than the processing of infected ducks. This finding may be due to greater infectivity, virulence, and pathogenicity (i.e., viral loads present systemically) in asymptomatic infected chickens than in ducks. Chickens are highly susceptible to HPAI viruses and in particular to Gs/GD lineage H5N1 viruses, which usually causes multiple organ failure associated with systemic virus replication and high mortality rates (*22*). By comparison, domestic ducks have shown moderate to high susceptibility to post-2002 Gs/GD H5N1 HPAI viruses (*23*). The lack of virus replication in duck endothelial cells and the absence of associated vascular damage has been identified as a key difference in pathogenesis between domestic ducks and chickens (*23**,**24*), which could determine not only the extent of replication for certain H5N1 HPAI viruses but also the quantities of virus found in different tissues. Previous reports have shown lower H5N1 virus titers in duck tissues than corresponding chicken tissues after intranasal inoculation (*25**–**27*). It is worth highlighting that age at infection can affect the pathogenicity of Gs/GD H5N1 HPAI viruses; VN/04 virus is more pathogenic and can replicate to higher titers (up to 4 log_10_ EID_50_/mL difference) in 2-week-old ducks than in 5-week-old ducks (*23**,**28*). The great majority of pathogenicity studies in domestic ducks use 2- to 5-week-old birds, whereas our studies required older ducks to match the age of slaughter in LPMs. The use of older ducks could have reduced the infectivity, replication, and virulence of H5N1 HPAI viruses, limiting systemic virus replication and reducing the quantity of virus incorporation into airborne particles generated during slaughter. Another factor responsible for differences between chickens and ducks is that the tested viruses were of chicken, human, and swan origin; whether a duck-origin virus would have been more efficient at generating infectious aerosols during duck manipulation needs to be investigated. In addition, all birds were confirmed to be infected at the moment of slaughter, but virus quantification in swab samples was not attempted; whether differences in oropharyngeal virus replication could explain differences in aerosolization and transmission is worth pursuing in future studies.

The slaughter of H5N1 virus–infected chickens had variable efficiency in producing infectious airborne particles and was not associated with specific HA genetic clades. However, specific changes in the HA and other gene segments could play a relevant role in airborne transmission. Similarly, sequence polymorphisms in internal proteins, in addition to those previously described for HA, may regulate airborne transmission of HPAI virus strains in mammals (*4**,**29*). Furthermore, the processing of A/chicken/Chile/184240–1(4322)/2002(H7N3) HPAI virus–infected chickens did not produce airborne virus (D.E. Swayne, unpub. data), compatible with the lack of human cases during the outbreak in Chile (*30*). However, human infections with H7N9 low pathogenicity AI virus have frequently been reported in China since 2013 (*31*), with a clear link between human cases and LPM exposure (*1**,**2**,**9*). These data suggest that not only H5N1 HPAI viruses have the potential to generate transmissible particles but also some H7 AI viruses (*4*) and potentially H9N2 viruses (*4*).

The LPM setting offers a variety of live bird species, providing an ideal environment to introduce and maintain AI viruses in the poultry population (*9*). Although intranasal administration is considered a standard practice for the study of AI virus pathogenicity, it is not the natural route of infection by contact or airborne routes. To our knowledge, this study is the closest re-creation of airborne transmission in the home or LPM slaughter setting. Naive chickens and ferrets exposed to the slaughter of Mong/05 and VN/04 virus–infected chickens, respectively, became infected and died. This finding confirms that the slaughter of infected chickens is an efficient source of exposure not only to other birds but also to ferrets, which are the model for human influenza transmission. The pathogenicity observed in chickens exposed to airborne Mong/05 was consistent with that observed in previous studies of systemic disease after intranasal inoculation of Gs/GD HPAI viruses (*22*). Similarly, the high pathogenicity and systemic infection in ferrets exposed to airborne VN/04 is consistent with that found by previous pathogenicity studies with this and other HPAI viruses in intranasally inoculated ferrets (*32**–**35*). Overall, these data confirm that the natural airborne route produces comparable infections to those produced by the commonly used intranasal route (*5**,**36**,**37*). Ocular exposure probably contributed to transmission because ocular mucosa represents a potential site for both replication and entry of airborne respiratory viruses (*38**–**40*).

In contrast, naive ducks and ferrets exposed to the same air space as the processing of VN/04 virus–infected ducks caused airborne infections in some of the animals. Although virus was isolated from swab samples of some exposed ducks, the lack of illness and death and lack of consistent seroconversion suggested that the slaughtering of infected ducks did not generate sufficient quantities of airborne viable virus to consistently produce infection in exposed ducks and failed to transmit virus to ferrets. Low levels of local replication at the mucosal level could have induced low levels of circulating antibodies in exposed ducks; therefore, systemic antibody titers may have been under the limit of detection. Collectively, these findings suggest that the processing of infected 8-week-old ducks may not be as consistent a source of airborne virus as processing infected chickens. One reason may be the age at slaughter: older ducks may not support such systemic virus replication as do chickens, lowering the quantities of generated airborne virus and, consequently, not reaching the minimum infectious dose required to efficiently infect naive adult ducks and ferrets. Another reason could be the lower number of slaughtered infected ducks (n = 5) compared with chickens (n = 10) per airborne exposure group, which implies a shorter exposure time for naive ducks.

In addition to the slaughter processes and the environmental conditions, time parameters were controlled to emulate field conditions (Table 2). Previous transmission studies in co-housed animals generally involve continuous exposure in which the recipient and donor animals are exposed to the same air space and sometimes fomites for 14 days (*41*). However, exposures of uninfected humans to others with seasonal influenza viruses are limited to a few hours (*42*), similar to HPAI virus exposure during slaughter or other manipulations of infected poultry. Each processing trial in our study lasted for < 1 h because of the need to mimic time-limited exposure events (*41*). Because this limit of exposure probably decreased successful transmission events compared with other animal studies with longer exposure times, we believe that our experiments more appropriately reflect the transmissibility of airborne AI viruses to humans and emphasize the high risk that slaughtering infected poultry entails (*41*). Although all the steps in the slaughter procedure may contribute to virus aerosolization, defeathering is often identified as a main risk activity (*4**,**12*). Further research to determine the most contaminat-ing steps will help develop efficient mitigating measures.

This study recreates generation and transmission of infectious influenza airborne virus particles by processing infected poultry in an experimental setting, matching time exposure events. We confirmed that the simulated slaughter of chickens infected with different clades of Gs/GD lineage H5N1 viruses generated infectious droplets and aerosols. Moreover, naive chickens and ferrets exposed to the same air space as the slaughter of infected chickens became infected and died, but the same could not be consistently confirmed following the slaughter of infected ducks. Further experiments investigating simple, feasible changes in slaughter methods to prevent or reduce infectious airborne particles during the slaughter process, and determining the effectiveness of such strategies on reducing virus transmission, are critical for preventing zoonotic HPAI (H5N1) virus infections of humans.

## References

[1] Cumulative number of confirmed human cases of avian influenza A(H5N1) reported to WHO. [cited 2017 Aug 23]. http://www.who.int/influenza/human_animal_interface/H5N1_cumulative_table_archives/en/.

[2] Lai S, Qin Y, Cowling BJ, Ren X, Wardrop NA, Gilbert M, et al. Global epidemiology of avian influenza A H5N1 virus infection in humans, 1997-2015: a systematic review of individual case data. Lancet Infect Dis. 2016;16:e108–18. 10.1016/S1473-3099(16)00153-5 27211899 PMC4933299

[3] Abdel-Ghafar AN, Chotpitayasunondh T, Gao Z, Hayden FG, Nguyen DH, de Jong MD, et al.; Writing Committee of the Second World Health Organization Consultation on Clinical Aspects of Human Infection with Avian Influenza A (H5N1) Virus. Update on avian influenza A (H5N1) virus infection in humans. N Engl J Med. 2008;358:261–73. 10.1056/NEJMra0707279 18199865

[4] Richard M, Fouchier RA. Influenza A virus transmission via respiratory aerosols or droplets as it relates to pandemic potential. FEMS Microbiol Rev. 2016;40:68–85. 10.1093/femsre/fuv039 26385895 PMC5006288

[5] Gustin KM, Belser JA, Wadford DA, Pearce MB, Katz JM, Tumpey TM, et al. Influenza virus aerosol exposure and analytical system for ferrets. Proc Natl Acad Sci U S A. 2011;108:8432–7. 10.1073/pnas.1100768108 21536880 PMC3100970

[6] Cowling BJ, Ip DK, Fang VJ, Suntarattiwong P, Olsen SJ, Levy J, et al. Aerosol transmission is an important mode of influenza A virus spread. Nat Commun. 2013;4:1935. 10.1038/ncomms2922 23736803 PMC3682679

[7] Milton DK, Fabian MP, Cowling BJ, Grantham ML, McDevitt JJ. Influenza virus aerosols in human exhaled breath: particle size, culturability, and effect of surgical masks. PLoS Pathog. 2013;9:e1003205. 10.1371/journal.ppat.1003205 23505369 PMC3591312

[8] Lindsley WG, Noti JD, Blachere FM, Thewlis RE, Martin SB, Othumpangat S, et al. Viable influenza A virus in airborne particles from human coughs. J Occup Environ Hyg. 2015;12:107–13. 10.1080/15459624.2014.973113 25523206 PMC4734406

[9] Suarez DL. Influenza A virus. In: Swayne DE, editor. Animal Influenza. Ames (IA): Blackwell Publishing; 2016. p. 3–30.

[10] Indriani R, Samaan G, Gultom A, Loth L, Irianti S, Adjid R, et al. Environmental sampling for avian influenza virus A (H5N1) in live-bird markets, Indonesia. Emerg Infect Dis. 2010;16:1889–95. 10.3201/eid1612.100402 21122218 PMC3294595

[11] Kang M, He J, Song T, Rutherford S, Wu J, Lin J, et al. Environmental sampling for avian influenza A(H7N9) in live-poultry markets in Guangdong, China. PLoS One. 2015;10:e0126335. 10.1371/journal.pone.0126335 25933138 PMC4416787

[12] Zhou J, Wu J, Zeng X, Huang G, Zou L, Song Y, et al. Isolation of H5N6, H7N9 and H9N2 avian influenza A viruses from air sampled at live poultry markets in China, 2014 and 2015. Euro Surveill. 2016;21:30331. 10.2807/1560-7917.ES.2016.21.35.30331 27608369 PMC5015459

[13] World Organisation for Animal Health. Avian influenza (infection with avian influenza viruses). In: Manual of Diagnostic Tests and Vaccines for Terrestrial Animals. 2015 [cited 2017 Aug 25]. http://www.oie.int/fileadmin/Home/fr/Health_standards/tahm/2.03.04_AI.pdf.

[14] Lindsley WG, Schmechel D, Chen BT. A two-stage cyclone using microcentrifuge tubes for personal bioaerosol sampling. J Environ Monit. 2006;8:1136–42. 10.1039/b609083d 17075620

[15] Lohren U. Overview on current practices of poultry slaughtering and poultry meat inspection. European Food Safety Authority Supporting Publications. 2012;EN-298 [cited 2017 Sep 6]. 10.2903/sp.efsa.2012.EN-298

[16] Swayne DE, Senne DA, Suarez DL. Avian influenza. In: Dufour-Zavala L, Swayne DE, Glisson JR, Pearson JE, Reed WM, Jackwood MW, et al., editors. Isolation and identification of avian pathogens. Jacksonville (FL): American Association of Avian Pathologists; 2008. p. 128–34.

[17] Perkins LEL, Swayne DE. Pathobiology of A/chicken/Hong Kong/220/97 (H5N1) avian influenza virus in seven gallinaceous species. Vet Pathol. 2001;38:149–64. 10.1354/vp.38-2-149 11280371

[18] Corzo CA, Culhane M, Dee S, Morrison RB, Torremorell M. Airborne detection and quantification of swine influenza a virus in air samples collected inside, outside and downwind from swine barns. PLoS One. 2013;8:e71444. 10.1371/journal.pone.0071444 23951164 PMC3738518

[19] Alonso C, Raynor PC, Davies PR, Torremorell M. Concentration, size distribution, and infectivity of airborne particles carrying swine viruses. PLoS One. 2015;10:e0135675. 10.1371/journal.pone.0135675 26287616 PMC4545937

[20] Torremorell M, Alonso C, Davies PR, Raynor PC, Patnayak D, Torchetti M, et al. Investigation into the airborne dissemination of H5N2 highly pathogenic avian influenza virus during the 2015 spring outbreaks in the midwestern United States. Avian Dis. 2016;60:637–43. 10.1637/11395-021816-Reg.1 27610723

[21] Lindsley WG, Green BJ, Blachere FM, Martin SB, Law BF, Jensen PA, et al. Sampling and characterization of bioaerosols. In: Ashley K, O'Connor PF, editors. NIOSH manual of analytical methods. 5th ed. Cincinnati (OH): National Institute for Occupational Safety and Health; 2017. p. BA1–115.

[22] Spickler AR, Trampel DW, Roth JA. The onset of virus shedding and clinical signs in chickens infected with high-pathogenicity and low-pathogenicity avian influenza viruses. Avian Pathol. 2008;37:555–77. 10.1080/03079450802499118 19023755

[23] Pantin-Jackwood MJ, Swayne DE. Pathobiology of Asian highly pathogenic avian influenza H5N1 virus infections in ducks. Avian Dis. 2007;51(Suppl):250–9. 10.1637/7710-090606R.1 17494561

[24] Kuiken T, van den Brand J, van Riel D, Pantin-Jackwood M, Swayne DE. Comparative pathology of select agent influenza a virus infections. Vet Pathol. 2010;47:893–914. 10.1177/0300985810378651 20682805

[25] Kishida N, Sakoda Y, Isoda N, Matsuda K, Eto M, Sunaga Y, et al. Pathogenicity of H5 influenza viruses for ducks. Arch Virol. 2005;150:1383–92. 10.1007/s00705-004-0473-x 15747052

[26] Jeong OM, Kim MC, Kim MJ, Kang HM, Kim HR, Kim YJ, et al. Experimental infection of chickens, ducks and quails with the highly pathogenic H5N1 avian influenza virus. J Vet Sci. 2009;10:53–60. 10.4142/jvs.2009.10.1.53 19255524 PMC2801098

[27] Suzuki K, Okada H, Itoh T, Tada T, Mase M, Nakamura K, et al. Association of increased pathogenicity of Asian H5N1 highly pathogenic avian influenza viruses in chickens with highly efficient viral replication accompanied by early destruction of innate immune responses. J Virol. 2009;83:7475–86. 10.1128/JVI.01434-08 19457987 PMC2708648

[28] Pantin-Jackwood MJ, Suarez DL, Spackman E, Swayne DE. Age at infection affects the pathogenicity of Asian highly pathogenic avian influenza H5N1 viruses in ducks. Virus Res. 2007;130:151–61. 10.1016/j.virusres.2007.06.006 17658647

[29] Linster M, van Boheemen S, de Graaf M, Schrauwen EJA, Lexmond P, Mänz B, et al. Identification, characterization, and natural selection of mutations driving airborne transmission of A/H5N1 virus. Cell. 2014;157:329–39. 10.1016/j.cell.2014.02.040 24725402 PMC4003409

[30] Max V, Herrera J, Moreira R, Rojas H. Avian influenza in Chile: a successful experience. Avian Dis. 2007;51(Suppl):363–5. 10.1637/7631-042806R1.1 17494584

[31] Gao R, Cao B, Hu Y, Feng Z, Wang D, Hu W, et al. Human infection with a novel avian-origin influenza A (H7N9) virus. N Engl J Med. 2013;368:1888–97. 10.1056/NEJMoa1304459 23577628

[32] Hulse-Post DJ, Franks J, Boyd K, Salomon R, Hoffmann E, Yen HL, et al. Molecular changes in the polymerase genes (PA and PB1) associated with high pathogenicity of H5N1 influenza virus in mallard ducks. J Virol. 2007;81:8515–24. 10.1128/JVI.00435-07 17553873 PMC1951362

[33] Lednicky JA, Hamilton SB, Tuttle RS, Sosna WA, Daniels DE, Swayne DE. Ferrets develop fatal influenza after inhaling small particle aerosols of highly pathogenic avian influenza virus A/Vietnam/1203/2004 (H5N1). Virol J. 2010;7:231. 10.1186/1743-422X-7-231 20843329 PMC2949836

[34] Giles BM, Ross TM. A computationally optimized broadly reactive antigen (COBRA) based H5N1 VLP vaccine elicits broadly reactive antibodies in mice and ferrets. Vaccine. 2011;29:3043–54. 10.1016/j.vaccine.2011.01.100 21320540 PMC3090662

[35] Belser JA, Tumpey TM. H5N1 pathogenesis studies in mammalian models. Virus Res. 2013;178:168–85. 10.1016/j.virusres.2013.02.003 23458998 PMC5858902

[36] Gustin KM, Katz JM, Tumpey TM, Maines TR. Comparison of the levels of infectious virus in respirable aerosols exhaled by ferrets infected with influenza viruses exhibiting diverse transmissibility phenotypes. J Virol. 2013;87:7864–73. 10.1128/JVI.00719-13 23658443 PMC3700211

[37] Belser JA, Gustin KM, Katz JM, Maines TR, Tumpey TM. Comparison of traditional intranasal and aerosol inhalation inoculation of mice with influenza A viruses. Virology. 2015;481:107–12. 10.1016/j.virol.2015.02.041 25771498 PMC5725743

[38] Bischoff WE, Reid T, Russell GB, Peters TR. Transocular entry of seasonal influenza-attenuated virus aerosols and the efficacy of n95 respirators, surgical masks, and eye protection in humans. J Infect Dis. 2011;204:193–9. 10.1093/infdis/jir238 21673029 PMC3164472

[39] Belser JA, Gustin KM, Maines TR, Pantin-Jackwood MJ, Katz JM, Tumpey TM. Influenza virus respiratory infection and transmission following ocular inoculation in ferrets. PLoS Pathog. 2012;8:e1002569. 10.1371/journal.ppat.1002569 22396651 PMC3291616

[40] Belser JA, Gustin KM, Katz JM, Maines TR, Tumpey TM. Influenza virus infectivity and virulence following ocular-only aerosol inoculation of ferrets. J Virol. 2014;88:9647–54. 10.1128/JVI.01067-14 24920819 PMC4136304

[41] Lakdawala SS, Subbarao K. The ongoing battle against influenza: The challenge of flu transmission. Nat Med. 2012;18:1468–70. 10.1038/nm.2953 23042349

[42] Killingley B, Enstone JE, Greatorex J, Gilbert AS, Lambkin-Williams R, Cauchemez S, et al. Use of a human influenza challenge model to assess person-to-person transmission: proof-of-concept study. J Infect Dis. 2012;205:35–43. 10.1093/infdis/jir701 22131338

